# Retrograde intra renal surgery (RIRS) versus percutaneous nephrolithotomy (PNL) as a primary treatment for large renal stones: A prospective randomized controlled trial

**DOI:** 10.1080/20905998.2025.2515354

**Published:** 2025-06-03

**Authors:** Ayman Kassem, Hesham Torad, Ahmed Essam, Mahmoud Abdel Hamid, Sameih Zamel, Amr Elkady

**Affiliations:** Kasr Alainy School of Medicine, Cairo University, Cairo, Egypt

**Keywords:** Flexible ureteroscopy, percutaneous nephrolithotomy, renal stones

## Abstract

**Background:**

Despite the high efficacy of percutaneous nephrolithotomy (PNL), it has more morbidity and a difficult learning curve. Retrograde intra renal surgery (RIRS) was introduced as a minimally invasive procedure for treatment of renal stones.

**Objective:**

To compare RIRS versus PNL in the management of renal stones sized 2–3 cm.

**Patient and methods:**

In this prospective randomized controlled trial, 122 patients with renal stones 2–3 cm were included and divided into two equal groups. Group A underwent RIRS. Group B underwent PNL. Patients with bleeding disorders, pregnancy, active UTI were excluded. Laboratory investigations, Ultrasound, CTUT were done preoperatively. Perioperative outcome (operative time, complications, hospital stay and pain score) was recorded. SFR (stone free rate) was assessed by CTUT after one month.

**Results:**

The mean stone size for group A and B were 2.11 ± 0.21 and 2.12 ± 0.23, respectively. The Stone free rate was 70.5% in RIRS and 73.8% in PNL (*p* = 0.840). lithotripsy time was significantly longer in the RIRS group (84.75 vs 72.95 minutes) (*p* = 0.019). PNL group showed significant post-operative hemoglobin drop but with no need for blood transfusion. High-grade fever was slightly higher in the RIRS group (8.2% vs 6.5%) (*p* = 0.557). Sepsis developed in 4.9% of RIRS group and 1.6% of the PNL group (*p* = 0.362). One case of mortality was reported in the PNL group. The hospital stay was significantly longer in the PNL group. The mean pain score was significantly lower in the RIRS.

**Conclusion:**

RIRS can be used as an alternative to PNL for the management of renal stones sized 2–3 cm, with comparable stone free rates, less hospital stays, less pain score, less hemoglobin drops. but longer lithotripsy time.

## Introduction

Urinary calculi are still a cause of significant morbidity despite the advances in the treatment options; with a prevalence rate varying from 1% to 20% [1]. Treatment is based on many parameters such as stone size, number, location and composition in addition to the anatomy of the pelvi-calyceal system.

Up till now, PNL is considered the standard treatment option for renal stones more than 2 cm according to the American Urological Association (AUA) guidelines, and the European association of urology (EAU) guidelines.

Despite the high efficacy of PNL defined as a better stone free rate rather than other alternatives, it has more morbidity [2] with more difficult learning curve and less acceptability from some patients to ‘puncture’ their kidneys.

A systematic review done by Seitz, C et al. and included 12,000 patients showed that the rates of complications of PNL were fever (10.8%), transfusion (7%), thoracic complications (1.5%), sepsis (0.5), organ injury (0.4%), embolization (0.4%), urinoma (0.2%), and death in 0.05% [2].

RIRS was introduced as an alternative for the treatment of renal stones with the advantage of being a less invasive procedure and acceptable stone free rates.

Major technological progress in RIRS has been achieved in the last few years. A systematic review addressing its efficacy in renal stones >2 cm showed a cumulative SFR of 91% with 1.45 procedures/patient; 4.5% of the complications were >Clavien 3 [3,4].

The aim of this study was to compare between RIRS and PNL in the management of renal stones from 2 to 3 cm.

## Patients and methods

### Study population

After institutional review board approval (IRB), (code: MD-299–2022) we recruited 122 patients with renal stones 2–3 cm, between october 2022 till August 2023. randomized using random number generator plus version 2.4.8 into two groups. (Group A) underwent RIRS and (Group B) underwent PNL. The inclusion criteria of patients included single renal stone from 2 to 3 cm in diameter, and radio-opaque or radio-lucent stones, pelvic or calyceal stones. Exclusion criteria were stone burden below 2 cm or above 3 cm, bleeding disorder, pregnancy, active urinary tract Infection and pelvic kidney precluding PNL.

**Figure 1.**
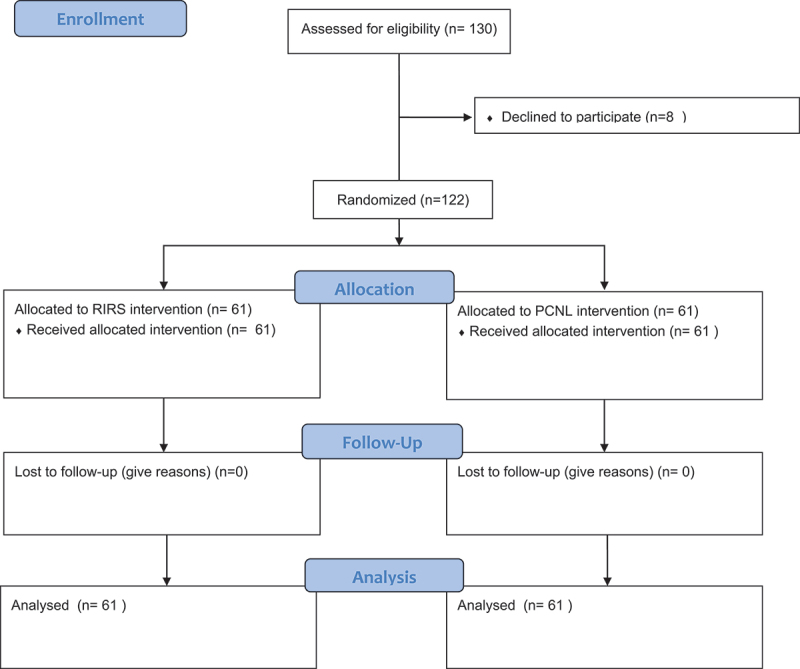
CONSORT flow Diagram.

Eligible patients were enrolled in the study after being informed about treatment options (Figure 1). An informed written consent was signed by all patients according to Good Clinical Practice and Declaration of Helsinki.

A detailed personal and urological history was attained. Pain was estimated using a pain score ranging 0–10, 0 when there is no pain, 1–3 for mild pain, while 4–6 for moderate pain, 7–9 for severe pain and 10 is the worst pain possible. Patients were asked to describe the degree of pain pre- and post-operatively.

A full physical examination with laboratory and radiological work up were done. Laboratory tests included CBC (complete blood count), renal function tests, liver function tests, coagulation profile, urine analysis, urine culture and sensitivity. Radiological investigations included Plain urinary tract x-ray (KUB), computerized tomography of the urinary tract (CTUT) was done to determine site, size, number and H.U. of stones.

All patients received preoperative prophylactic antibiotic Group (A); RIRS performed in lithotomy position. A guide wire (0.038 inches) was passed up to the kidney under fluoroscopic guidance, then sequential ureteral dilatation up to 14 F and a ureteral access sheath (11–13 F) was placed. (suction access sheath was not available at our institute) Ureteroscope (OTU 8.6 fr) passed through the access sheath up to the kidney for dusting the stones using holmium laser fiber 272 mm, with setting frequency 20 hZ and energy 0.5 J. At the end of the operation a DJ ureteric stent was fixed. And an alpha blocker was prescribed on discharge, to control stent-related irriative symptoms. CTUT was done after 1 month to detect any residual stone fragments (more than 2 mm).

Group (B); PNL performed in prone position, after passing guide wire and ureteric catheter in lithotomy position. Puncture the kidney, pass 2 wires, then use renal dilators and access sheath to enter the kidney by nephroscopy (Karl Storz). stone fragmentation by pneumatic lithotripsy and remove stones, then place nelaton 28F at end of operation. CTUT was performed after 1 month to detect presence of any residual stone fragments (more than 4 mm).

In both groups, we assessed operative time (from introduction of the endoscope till the end of the procedure), lithotripsy time, hospital stay, complications (fever, significant hematuria, renal pelvic perforation, colonic injury, urinary leakage, residual stones and the need for second look PNL or DJ fixation).

**Primary Outcome**: stone free rate between both groups

**Secondary Outcome**: hospital stay, complications, and pain score.

Data were coded and entered using the statistical package SPSS version 21. Data were summarised using number and the percent for qualitative variables and mean and standard deviation for quantitative variables. Comparison between groups were done using Chi Square test and Fishers exact test for qualitative variables, independent sample t test for quantitative variables which were normally distributed while non parametrical Mann Whitney test was used for quantitative variables which were not normally distributed. Correlations we’re done to test to four linear relations between variables. P-values less than or equal to 0.05 were considered as statistically significant.

### Sample size calculation

Using Epicalc 2000 version 1.02 with two-sided significance level (1-alpha): 95, Power 80%. Ratio of sample size, RIRS/ PNL:1. According to (RIRS versus PNL as primary treatment in renal stones 2 cm or greater [5]. Percent of stone free rate among RIRS 63%. Percent of stone free rate among PNL 85%. The required sample size in each group was 61.

## Results

The current study included a total of 122 patients with single renal stone 2–3 cm who were randomly assigned into two arms: RIRS and PNL, with 61 cases in each arm.

The mean age of patients in RIRS arm was 44.02 years, while in PNL arm, it was slightly higher at 47.26 years and 70.5% of the patients in RIRS arm were male, in comparison to 73.8% in PNL arm with no significant difference (Table 1)

**Table 1.** Demographic data, medical and surgical history of study groups.

		RIRS *n* = 61	PNL *n* = 61	p-value
Age (years)	Mean ± SD	44.02 ± 13.18	47.26 ± 12.97	0.113
Gender*	Male	43 (70.5%)	45 (73.8%)	0.645
	Female	18 (29.5%)	16 (26.2%)	
DM*		6(9.8%)	8(13.1%)	0.776
HTN*		6(9.8%)	10(16.4%)	0.421
HCV*		3(4.9%)	0(0.0%)	0.242
CKD*		3(4.9%)	2(3.3%)	1.000
IHD*		3(4.9%)	1(1.6%)	0.611
Hypothyroidism*		1(1.6%)	0(0.0%)	1.000
History of DVT*		1(1.6%)	0(0.0%)	1.000
History of Stroke*		1(1.6%)	0(0.0%)	1.000
Interstitial Lung Fibrosis*		0(0.0%)	1(1.6%)	1.000
Leukemia*		0(0.0%)	1(1.6%)	1.000
Recurrent stone former *		11(18.0%)	18(29.5%)	
Surgical history	Pyelolithotomy*	4(6.6%)	10(16.4%)	0.156
	PNL*	11(18.0%)	10(16.4%)	1.000
	URS*	3(4.9%)	8(13.1%)	0.206
	ESWL*	9(14.8%)	6(9.8%)	0.581
	Cystolithotomy*	3(4.9%)	1(1.6%)	0.611
	Nephrectomy*	0(0.0%)	1(1.6%)	1.000
	Open Stone Bladder*	1(1.6%)	0(0.0%)	1.000

*Data are given in Number (%).

The medical histories and surgical history were comparable in both arms (Table 1). The mean stone size and the Hounsfield unit (HU) values were also comparable in both arms, with no significant differences. Most of the stones in both groups were radio-opaque, and the distribution of stones by side (left vs. right) was comparable with no significant differences (Table 2).

**Table 2.** Stones and interventional-related data among study groups.

		RIRS *n* = 61	PNL *n* = 61	p-value
Stone size (cm) (Mean ± SD)		2.11 ± 0.21	2.12 ± 0.23	0.901
HU_value (Mean ± SD)		817.10 ± 199.31	803.93 ± 238.66	0.722
Stones Side (number%)	Left	26(42.6%)	35(57.4%)	0.147
	Right	35(57.4%)	26(42.6%)	
Hydroneohrosis (number%)	Mild	33(54.1%)	38(62.3%)	0.635
	Moderate	3(4.9%)	3(4.9%)	
	No	25(41.%)	20(32.8%)	
Stone free rate (number%)		43(70.5%)	45(73.8%)	0.840
Operative time (min) (Mean ± SD)		100.25 ± 33.88	102.54 ± 35.20	0.777
lithotripsy time (min) (Mean ± SD)		84.75 ± 33.50	72.95 ± 34.48	0.019*
Haemoglobin (g/dl) (Mean ± SD)	Pre	12.35 ± 1.34	12.38 ± 1.46	0.928
	Post	12.32 ± 1.34	11.59 ± 1.44	<0.001*
	Hg change	0.03 ± 0.18	0.79 ± 0.25	<0.001*
Pain score		5.52 ± 0.62	8.26 ± 0.66	<0.001*
Hospital stay(hours) (Mean ± SD)		28.33 ± 17.80	64.92 ± 89.09	<0.001*

*for significant *p* value (<0.05).

Stone free rate was slightly higher in PNL (73.8%) than in RIRS (70.5%) with no significant difference (*p* = 0.840). The mean operative time was also comparable, with 100.25 minutes in RIRS group and 102.54 minutes in PNL group (*p* = 0.777). However, lithotripsy time was significantly longer in RIRS group (84.75 minutes) compared to the PNL group (72.95 minutes) (*p* = 0.019). lithotripsy time was estimated from the introduction of the pneumatic lithotripter in PNL and laser fibre in RIRS group till the end of the operation, pre-operative haemoglobin levels were comparable between RIRS and PNL groups, with no significant difference (*p* = 0.928). However, a significant decrease in post-operative hemoglobin was noticed in PNL arm, with a mean of 11.58 g/dl compared to 12.32 g/dl in RIRS arm (*p* < 0.001). This difference was further reflected in hemoglobin change, where the PNL arm experienced a more substantial decrease, with a mean change of 0.79 g/dl compared to just 0.03 g/dl in RIRS arm (*p* < 0.001). a significantly lower mean pain score was noticed in RIRS arm, with a mean of 5.52, compared to mean score of 8.26 (*p* < 0.001) in PNL arm, (Table 2). Incidence of complications was higher in PNL arm where extravasation (3.3%), perforation (8.2%), subcutaneous hematoma (3.3%), and urinary leakage (8.2%) were observed only in the PNL group, (urinary leakage treated conservatively in 4 cases (6.6%) and one case of urinary leakage required DJ fixation (1.6%).) while none of these complications were encountered in the RIRS group (Table 3).

**Table 3.** Complications and Clavien-Dindo classification among study groups.

		RIRS *n* = 61	PNL *n* = 61	*p*-value
Perforation of renal pelvis		0 (0.0%)	5 (8.2%)	0.0001*
Subcutaneous hematoma		0(0.0%)	2(3.3%)	0.0001*
Urinary leakage		0(0.0%)	5(8.2%)	0.0001*
Colonic injury		0(0.0%)	1(1.6%)	0.0001*
High grade Fever		5(8.2%)	4 (6.6%)	0.557
Sepsis		3(4.9%)	1(1.6%)	0.362
Clavien-Dindo classification	I	59(96.7%)	48(78.7%)	*p* = 0.009*
	II	2(3.3%)	12(19.7%)	
	III	0 (0%)	1 (1.6%)	
	V	0(0%)	1(1.6%)	

*for significant *p* value (<0.05).

In terms of secondary outcomes, a significantly longer hospital stay was noticed in PNL group, with a mean of 64.92 hours compared to 28.33 hours in RIRS group (*p* < 0.001). The incidence of high-grade fever was slightly higher in RIBS group (8.2%) than in PNL group (6.6%), with no significant difference (*p* = 0.557). Similarly, sepsis was rare in both groups, but higher in RIRS, with no significant difference (*p* = 0.362). The mortality rate was low, with one death occurring in the PNL group (1.6%), (This patient had colonic injury and stool leakage in the abdomen. Exploration was done twice but ended up with septic shock and death) and none in the RIRS group, Overall, while hospital stay differed significantly, other secondary outcomes were comparable in both arms.

In RIRS arm five cases needed DJ stent fixation before RIRS due to tight ureter and were re-operated after 2 weeks.DJ fixation was fixed postoperatively in all cases of the RIRS group, but we had to fix DJ stent in 6 cases in the PNL group (1 case due to urinary leakage, 2 cases due to residual stones, 3 cases due to renal pelvis perforation)

The majority of patients in the RIRS arm (96.7%) experienced Grade I complications (the majority of these complications were stent related irritative symptoms), significantly higher than in the PNL arm (78.7%). Conversely, Grade II complications were more common in PNL arm (19.7%) compared to (3.3%)in RIRS arm. One patient (1.6%) in the PNL arm experienced a Grade V complication (Table 3).

Significant correlations were found between stone size and operation time across all groups (*p* < 0.001 for all subjects and RIRS, *p* = 0.011 for PNL). lithotripsy time strongly correlated with operation time (*r* > 0.975, *p* < 0.001 in all groups). For RIRS, total energy and pain score (VAS) also showed significant correlations with operation time (*p* < 0.001). No significant correlation was observed between operation time and other factors such as age, HU value, number of stones, serum creatinine, or haemoglobin levels (Table 4).

**Table 4.** Correlation between operation time and study parameters.

	All subjects		RIRS		PNL	
	R	P Value	R	P-Value	R	P-Value
Age (years)	0.043	0.639	0.008	0.953	0.070	0.593
Stone size (cm)	0.338	<0.001*	0.356	<0.001*	0.323	0.011*
HU_value	0.014	0.879	0.105	0.421	−0.057	0.662
Serum creatinine (mg/dl)	−0.016	0.860	−0.121	0.352	0.179	0.167
lithotripsy time (min)	0.975	<0.001*	0.995	<0.001*	0.998	<0.001*
Total energy in RIRS	–	–	0.979	<0.001*	–	–
Pain score (VAS)	0.152	0.095	0.492	<0.001*	0.104	0.424
Hospital stays (day)	−0.013	0.883	0.114	0.381	−0.055	0.672
Pre hemoglobin (g/dl)	0.052	0.571	0.021	0.870	0.078	0.550

## Discussion

Shock wave lithotripsy (SWL), PNL and RIRS are the main treatment modalities for renal stones. While PNL efficacy is hardly affected by the stone size, the stone free rates (SFRs) after SWL or RIRS are inversely proportional to stone size [6–8].

The aim of management of renal stones is to achieve stone free status with the least number of interventions using with the least invasive method with minimal complications.

Endourology (RIRS, PNL) had been introduced as an alternative for SWL because of the reduced need for multiple sessions and shorter time to achieve a stone-free status [9,10].

Up till now, guidelines recommend that: Stones size >20 mm should be managed primarily by PNL, Although PNL achieves a good stone free rate, but it has more serious complications. A systematic review done by Seitz, C et al. and included 12,000 patients showed that the rates of complications of PNL were fever (10.8%), transfusion (7%), thoracic complications (1.5%), sepsis (0.5), organ injury (0.4%), embolization (0.4%), urinoma (0.2%), and death in 0.05% [2].

EAU guidelines recommend RIRS (even for stones >2 cm) in cases where PNL or SWL are not an option. However, in this case, there is a higher need for a follow-up procedure and ureteric stent insertion. (strong recommendation, EAU 2024)

Based on the previous knowledge we tried to assess the outcome of RIRS in management of renal stones sized 2–3 cm compared to gold standard PNL.

In a meta-analysis done by Chen et al. who Pooled their data from 19 studies involving 1822 patients and compared clinical safety and efficacy of PNL versus RIRS in treatment of upper urinary tract calculi. They found that the stone-free rate of PNL group was significantly higher than that of RIRS group [11].

In our study, the stone-free rate (SFR) of PNL group was higher than that of RIRS, but the difference was statistically insignificant. Our explanations for the narrow gap in the SFR between both procedure, are small sample size, improved learning curve in RIRS especially with the use of disposable flexible ureteroscope, decreased experience of PNL especially in junior urologists with wide spread of RIRS as an alternative, improving laser lithotripsy devices, using dormia basket 0 tip in RIRS, use of access sheath in all cases and insertion of DJ ureteric stent in all case of RIRS and assessing stone free status at one month postoperatively as imaging performed early after treatment carries a high false positive results from dust or residual fragments, which may pass spontaneously [12].

In agreement with our study, Chen et al. found that the decline of hemoglobin and the need for blood transfusion in PNL were significantly higher than that of RIRS. Operation time, fever, infection and perforation rates were not significantly different between two surgical procedures. However, they did not compare hospital stay in both groups, and did not report other complications as organ injury, subcutaneous hematoma, and death rates as our study.

Pieras et al. evaluated 108 patients divided into two equal groups. comparing PNL with RIRS in renal stones of 2–3 cm. They found no differences in the stone-free rate between the two surgical techniques or in the complications such as infection, fever or urinary fistula. These findings agree with our study. However, in their study, significantly longer operative time was noted in the PNL group than in the RIRS group, which may be attributed to the use of 24F sheath in PNL in their study in comparison to 30F. sheath in our study [13]

Guangda et al. retrospectively evaluated 142 patients with multiple nephrolithiasis and compared flexible ureteroscopy (RIRS) with mini-percutaneous nephrolithotomy in their management [14]. They concluded that, for multiple renal stones within 1–2 cm (< or = 3), RIRS can achieve the same stone clearance as PNL, with shorter hospital stay, shorter operative time with fewer complications. These results agree with our study. However, they found that there was no significant difference between both procdures when the number of stones was more than 3. These findings were recently noticed by Elnahas et al. who found that multiple stones and FURS performed by non-experienced surgeons are associated with risk of missing trifecta of RIRS (stone free status in one session without complications) [15]

Cheng Y et al. compared safety and efficacy of (RIRS) vs. (PNL) in the management of isolated kidney stones in 99 patients divided into two groups, RIRS group (*n* = 48) and PNL group (*n* = 51) [16]. They found that stone free rate was significantly higher in PNL than in RIRS, in their study, PNL had significantly shorter operative time than RIRS in contrast to our study. Which may be attributed to different laser settings in RIRS groups. Additionally, in our study we used ureteral access sheath in all cases of RIRS to decrease operative time and intrarenal pressure and hence decrease the infectious complications.

In agreement with our study, PNL group had significantly longer hospital stay than RIRS group in Cheng Y et al. Which may be explained by less postoperative complications, less need for analgesic in RIRS and post-operative DJ insertion in all cases of RIRS. The incidence of postoperative fever, pleural injury and pneumothorax were comparable in both arms.

In 2019, a meta-analysis done by Zewu et al. included (657 patients) and compared retrograde flexible ureteroscopy (RIRS) with percutaneous nephrolithotomy (PNL) in treatment of renal stones (2–3 cm) [17]. found that PNL had a higher stone free rate than RIRS. But this difference was statistically insignificant. between RIRS and PNL (*p*- value 0.08.). The PNL group was associated with a higher rate of overall minor and intra-operative complications and longer hospital stay. These results matched our study.

In 2023, Cosmin c et al., compared retrograde flexible ureteroscopy (RIRS) and percutaneous nephrolithotomy in 250 patients with large renal solitary stones. (2–4 cm) [18]. They found that SFR was higher in PNL 90.4⁒ than in RIRS 68⁒which may be due to larger size of stones, Hospital stay was higher in PNL than RIRS. As in our work. The overall rate of complications was higher in RIRS than PNL. But major complications were more in PNL. As noted in Cosmin et al. findings, extending criteria regarding stone size in RIRS should be done cautiously because it is associated with decrease in SFR and increased complications.

In 2022, Mahmoud et al. found that there were no statistically significant differences in operative time between mini-PNL and RIRS groups. While fluoroscopy time and hospital stay were significantly shorter in the RIRS group than in the mini-PNL group in his study. Similarly, Hospital stay was significantly shorter in our study in the RIRS group compared to PNL [19].

Despite the high safety of RIRS, the main remaining challenge is the stone free rate with large renal stones, a systematic review and meta-analysis evaluating residual stone fragments following any treatment modality has demonstrated that reintervention should be scheduled for residual fragments larger than 4 mm [20]. in addition to increasing the stone burden in RIRS necessitates insertion of ureteric stent with stent-related symptoms

Based on our results we think RIRS is a good alternative for patients with 2–3 cm renal stones with shorter hospital stay and faster recovery. And less analgesic use. Continued research and clinical attention should focus on optimizing energy usage during stone fragmentation to improve procedural efficiency and outcomes. And therefore, extending the criteria of RIRS to include large renal stones >3 cm.

Future studies with long term follow up, are needed to better define the long-term outcomes and cost-effectiveness of RIRS versus PNL, particularly in terms of stone recurrence, effect on renal function and renal micro anatomy

limitation of our study is the small sample size. Additionally, lack of estimated cost of both procedures, lack of long term follows up to detect late complications and lack of estimation of influence of pelvic analyceal system anatomy on the results of both procedures.

## Conclusion

RIRS can be used as an alternative to PNL in management of renal stones from 2 to 3 cm, with shorter hospital stay, less pain score and less serious complications with non-inferior stone free rate.
